# PALLIA‐10, a screening tool to identify patients needing palliative care referral in comprehensive cancer centers: A prospective multicentric study (PREPA‐10)

**DOI:** 10.1002/cam4.2118

**Published:** 2019-05-04

**Authors:** Yann Molin, Caroline Gallay, Julien Gautier, Audrey Lardy‐Cleaud, Romaine Mayet, Marie‐Christine Grach, Gérard Guesdon, Géraldine Capodano, Olivier Dubroeucq, Carole Bouleuc, Nathalie Bremaud, Anne Fogliarini, Aline Henry, Nathalie Caunes‐Hilary, Stéphanie Villet, Christine Villatte, Véronique Frasie, Valérie Triolaire, Véronique Barbarot, Jean‐Marie Commer, Agnès Hutin, Gisèle Chvetzoff

**Affiliations:** ^1^ Léon Bérard cancer center Lyon France; ^2^ Cancer Institute of Montpellier Montpellier France; ^3^ Direction of Clinical Research and Innovation Cancer center Léon Bérard Lyon France; ^4^ François Baclesse Cancer Centre Caen France; ^5^ Bergonié Cancer Institute Bordeaux France; ^6^ Paoli‐Calmettes Institute Marseille France; ^7^ Jean Godinot Cancer Institute Reims France; ^8^ Curie Institute Paris France; ^9^ George‐François Leclerc Cancer Center Dijon France; ^10^ Antoine Lacassagne Cancer Center Nice France; ^11^ Cancer Institute of Lorraine – Alexis Vautrin Nancy France; ^12^ IUC Toulouse Oncopole Toulouse France; ^13^ Oscar Lambret Cancer Center Lille France; ^14^ Jean Perrin Cancer Center Clermont Ferrand France; ^15^ Paul Strauss Cancer Center Strasbourg France; ^16^ Curie Institute Saint Cloud France; ^17^ West Cancer Institute Saint Herblain René Gauducheau Center Nantes France; ^18^ Paul Papin Cancer Center Angers France; ^19^ Eugène Marquis Cancer Center Rennes France

**Keywords:** advanced cancer, decision making, palliative care, prognosis, quality of life, surveys and questionnaire

## Abstract

**Purpose:**

The identification and referral of patients in need of palliative care should be improved. The *French society for palliative support and care* recommended to use the PALLIA‐10 questionnaire and its score greater than 3 to refer patients to palliative care. We explored the use of the PALLIA‐10 questionnaire and its related score in a population of advanced cancer patients.

**Methods:**

This prospective multicentric study is to be conducted in authorized French comprehensive cancer centers on hospitalized patients on a given day. We aimed to use the PALLIA‐10 score to determine the proportion of palliative patients with a score >3. Main secondary endpoints were to determine the proportion of patients already managed by palliative care teams at the study date or referred to palliative care in six following months, the prevalence of patients with a score greater than 5, and the overall survival using the predefined thresholds of 3 and 5.

**Results:**

In 2015, eighteen French cancer centers enrolled 840 patients, including 687 (82%) palliative patients. 479 (69.5%) patients had a score >3, 230 (33.5%) had a score >5, 216 (31.4%) patients were already followed‐up by a palliative care team, 152 patients were finally referred to PC in the six subsequent months.

The PALLIA‐10 score appeared as a reliable predictive (adjusted OR_R__ef≤3_: 1.9 [1.17‐3.16] and 3.59 [2.18‐5.91]) and prognostic (adjusted HR_R__ef≤3_ = 1.58 [95%CI 1.20‐2.08] and 2.18 [95%CI 1.63‐2.92]) factor for patients scored 4‐5 and >5, respectively.

**Conclusion:**

The PALLIA‐10 questionnaire is an easy‐to‐use tool to refer cancer inpatients to palliative care in current practice. However a score greater than 5 using the PALLIA‐10 questionnaire would be more appropriate for advanced cancer patients hospitalized in comprehensive cancer center.

## 1 INTRODUCTION

In spite of steady improvements in anti‐cancer therapies in the past decades, in 2012 more than eight million cancer deaths were reported worldwide by the *Internal Agency for Research on Cancer* and the incidence rates for all cancer are still increasing.^1^, ^2^ Even though the disease per se cannot be treated even with the best available treatment modalities, a better control of cancer progression is achieved and more patients with metastatic disease live longer. This strengthened the growing need for supportive and palliative care (PC).

Based on evidence from randomized clinical trials, the international medical societies supported a greater integration of PC for cancer patients, therefore promoting PC referrals early in the disease trajectory, ie concomitant to the administration of specific treatments.^3^, ^4^, ^5^, ^6^ Smith et al recommended in 2012 simultaneous curative and palliative support for patients in metastatic setting, or presenting severe symptoms,^7^ and the integration of PC into oncology clinical practice was adopted by ASCO in 2017.^6^, ^7^ Reduction in symptoms including depressive symptoms, and an improved quality of life were reported in patients receiving early PC.^8^, ^9^, ^10^, ^11^ A concomitant intervention of the medical oncology and the PC teams showed a prolonged 1‐year survival for patients having received concomitant intervention (63%) vs no PC intervention (48%),^9^ and improved referrals of advanced patients nearing the end of life.^9^, ^12^, ^13^

An improved integration of PC in the global care management should be expected in the specific context of comprehensive cancer centers. However, the current size of PC teams does not allow to cope with the increasing demands for support. In order to improve global patient care, medical oncology teams need to identify the patients who require priority benefit from concomitant PC. The consensual segmentation with successive stages throughout the continuum of the disease proposed by Krakowski and colleagues is widely used in France to schedule the PC intervention.^14^ Prognostic scores would be helpful in the clinical decision‐making process, however, their implementation is challenging; some well‐known instruments including the Palliative Prognostic Score (PaP) have been validated in patients with cancer.^15^, ^16^, ^17^, ^18^, ^19^, ^20^, ^21^, ^22^, ^23^ Scoring methods initially developed for home hospice or palliative care setting are more adapted to patients with a short prognostic value typically measured in days to weeks. These scores were most often developed to predict short‐term mortality and not specifically to assist in identifying a need for PC intervention.^24^ The recent Pronopall score has been proposed to better characterize ambulatory or hospitalized patients with distinct prognosis.^23^ Unfortunately, these prognostic estimations do not accurately reflect the complexity of situations, and the prognostic scores are often considered as too restrictive.^18^, ^25^, ^26^ Faced with such shortcomings, the health care teams were not convinced by the proposed tools who rarely use these questionnaires in daily practice. Indeed, prognosis‐based criteria may be less appropriate than need‐based criteria.^27^, ^28^

An international consensus recently suggested not to use instruments but rather a combination of major and minor criteria to identify outpatients to be referred to specialized palliative care, as earlier proposed for in‐patients.^29^

The National Comprehensive Cancer Network (NCCN) proposed a two‐step screening process that was demonstrated to be feasible and a simplified version was then evaluated.^30^, ^31^, ^32^ In parallel, the *French society for palliative support and care* (SFAP) proposed in June 2010 a multidimensional questionnaire addressing medical, psycho‐social and ethical issues. This 10‐item screening form PALLIA‐10 designed to be used by any caregiver, aimed to easily assign to patients a score from 0 to 10 (Supplementary materials p1,2). The PALLIA‐10 questionnaire is an alternative scale intending to discriminate patients on their PC requirement; its relevance still warrant to be assessed, and the correlation between the use of the questionnaire, PC interventions, and survival has not been established so far. The current recommendations aim to refer any patient with a PALLIA‐10 score >3 to a dedicated PC team. However, comprehensive cancer centers usually provide care to patients with advanced disease already heavily treated, late in the course of the disease. An improved selection procedure is required to trigger the intervention of palliative care teams. This prospective multicentric study implemented the use of the PALLIA‐10 questionnaire in advanced cancer patients hospitalized in comprehensive cancer centers and explored the use of a threshold at 3 to appropriately refer patients to palliative care.

## 2 METHODS

### 2.1 Patients and study procedures

This prospective study enrolled hospitalized adult patients in conventional medicine or in radiotherapy departments in 18 of the 20 French comprehensive cancer centers. Patients in surgery departments and outpatients were excluded.

Eligible participants and family caregivers if applicable, received oral and written information about the study and were free to opt out of the study. The study received approval of the French advisory committee on information in health research (CCTIRS) and the national commission for informatics and rights (CNIL), was notified to the Ethic committee of Lyon Sud‐Est IV and was registered on ClinicalTrials.gov, number NCT02479061.

Descriptive data (age, gender, familial status, and Karnofsky index translated to Eastern Cooperative Oncology Group‐Performance Status (ECOG‐PS), disease characterization (diagnosis, setting), and the global medical care management (reason for hospitalization, PC requirement) were collected. To note, the most recent biological data collected within a maximum of 3 weeks from enrollment were used.

The PALLIA‐10 questionnaire was intended to be exclusively filled out for patients with incurable disease on the basis of the current knowledge. A single national site initiation meeting was organized to inform all participating centers on specific procedures for enrollment, questionnaire completion guidelines and data collection. Each investigational team involving one physician and at least one nurse from each supportive care unit, was specifically informed on how to use the PALLIA‐10 questionnaire, and how to assign a PALLIA‐10 score ranging from a scale from 0 to 10 based on the SFAP recommendations. To note, the SFAP mentioned that any person should be able to complete the questionnaire regardless its professional background. Patients or their caregiver if applicable were directly questioned. The recruitment was performed in each institution on a single day to ensure a single evaluation per bed. The setting of the disease, either curative, early palliative, terminal palliative, or agonic stage was assessed according to the Krakowski classification.^14^

Survival data were updated 6 months after the study for all patients, and intercurrent date of PC initiation reported for patients not already managed by a palliative care team at the date of the study, when applicable.

### 2.2 Outcomes

We determined the prevalence, the decision for PC intervention adopted and the overall survival for palliative patients with a PALLIA‐10 score greater than 3 in the population of patients hospitalized in a French cancer center, in parallel with the PALLIA‐10 predefined thresholds of 3 and then 5. We also assessed the proportion of patients already managed by a PC in the global population, in patients with a PALLIA‐10 score greater than 3 and 5, the median PALLIA‐10 score in patients already followed‐up by a PC team.

### 2.3 Statistical analysis

We considered the palliative patient population for the analysis. A palliative entry date allowed classification of the disease as palliative, otherwise the assessment of the medical oncologist based on Krakowski classification was considered.^14^ Descriptive statistics were used to summarize patients’ initial characteristics at inclusion. Overall survival (OS) was estimated from the date of inclusion to the date of death from any cause or censored at the date of the last follow‐up. Survival curves for OS with associated log‐rank tests were generated using the Kaplan Meier method. The thresholds of 3 and 5 for the PALLIA‐10 score were defined before the analysis.

A Cox proportional hazards model was performed to adjust PALLIA‐10 score (≤3*,* 4‐5, and >5) for confounding factors. The variables considered were age at inclusion, reason for hospitalization, type of tumors, PC management at the time of inclusion, the convergence of opinions between the oncologist, the healthcare team and the PC team, Karnofsky score, number of metastatic sites, and biological parameters (hemoglobin, lymphocytes, LDH, albumin, and CRP). Variables sufficiently informed (less than 10% missing values) and significant at a 20% level were included in a backward selection procedure to keep factors significant at a 5% level in the final multivariate model. We also performed a multivariate Cox model taking into account PALLIA‐10 score in quantitative. Hazard Ratios (HRs) are presented with 95% confidence intervals (CI).

We performed a logistic regression analysis to evaluate the correlation of the following pre‐specified factors to PC management in two steps, firstly PALLIA‐10 score (≤3, and >3), and further explored PALLIA‐10 score (≤3*,* 4‐5, and >5), familial status, age at inclusion, reason for hospitalization, type of tumor, the opinion convergence between oncologist, healthcare team and PC team, Karnofsky score, number of metastatic sites, and biological parameters (hemoglobin, lymphocytes, LDH, albumin, and CRP). Variables sufficiently informed (less than 10% missing values) and significant at a 20% level were included in a backward selection procedure to keep factors significant at a 5% level in the final. Odd Ratio (OR) are presented with 95% confidence interval (CI).

We plotted a receiver operator characteristic (ROC) analysis to examine the sensitivity and specificity of the PALLIA‐10 scores. The Youden index J was calculated to determinate the cut‐off point for optimal sensitivity and specificity. A *P*‐value <0.05 was considered statistically significant. SAS version 9.4 was used for all statistical analyses.

## 3 RESULTS

**Figure 1:**
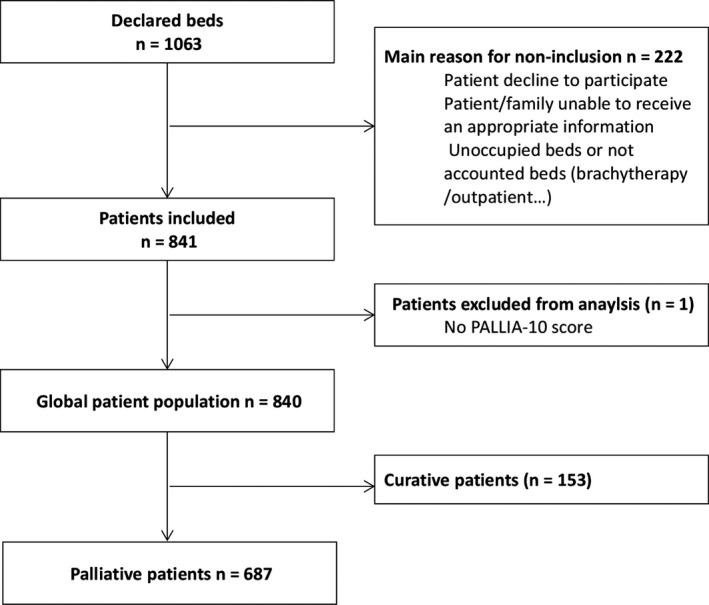
Trial profile. Reasons for non‐inclusion have not been quantified

The study was conducted in 18 (90%) French Comprehensive Cancer Centers. Sixteen centers carried out this prospective data collection on a single day each, in 2015, from Jun 15th to 19th, and two additional sites on Oct 8th and Oct 9th (Supplementary materials p3). The participating sites declared an overall number of 1063 hospital beds. After exclusion of minors patients, outpatient hospitalizations, and brachytherapy or unoccupied beds, 841 patients were considered and 687 (82%) were in palliative setting (Figure 1). To note, the PALLIA‐10 score was missing for one patient.

The median age at inclusion was 64.8 (19‐92), 371 (54.0%) patients were women, and 490 (71.3%) patients were living with a caregiver. 587 (85.4%) patients had a PS ≥ 2. The most frequent localizations of the primary disease were digestive tract for 126 (18.3%) patients, breast for 121 (17.6%) patients, and lung and/or pleura for 101 (14.7%) patients. Metastatic sites were identified for 599 (87.4%) patients, including more than two metastatic sites for 69.3% of patients. The patients’ characteristics are summarized in Table 1.

**Table 1:** Main demographics and baseline characteristics. Data are median (range min‐max) or *n* (%)

	Patients in palliative setting *N* = 687
Median age at inclusion (years)	64.8 (19‐92)
Median age at diagnosis (years)	61.6 (12‐90)
Gender	
Female	371 (54%)
Male	316 (46%)
Familial status	
Missing data	1
Alone	167 (24.3%)
Caregiver at home^a^	490^a^ (71.4%)
Dependent person at home	29 (4.2%)
Performance Status (ECOG‐PS)	
0	5 (0.7%)
1	95 (13.8%)
2	202 (29.4%)
3	210 (30.6%)
4	175 (25.5%)
Main primary tumor localizations^a^	
Digestive tract	126 (18.3%)
Breast	121 (17.6%)
Lung and/or pleura	101 (14.7%)
Head and Neck	63 (9.2%)
Urologic	82 (11.9%)
Gynaecologic	78 (11.4%)
Metastatic disease	
Missing data	2
Metastatic disease	599 (87.4%)
Median number of metastatic sites	2.0 (0‐8)
Reason(s) for hospitalization	
Treatment (chemotherapy, radiotherapy…) or health assessments	212 (30.9%)
Acute complication (aplasia, sepsis, Intracranial hypertension…)	137 (20.0%)
Symptoms (pain, dyspnea…)	338 (49.2%)
Median delay between hospitalization date and inclusion (days)	6.0 (0‐77)
Patients with entry date in palliative setting	668 (97.2%)
Median delay between initial diagnosis and palliative setting^a^ (months)	3.0 (0‐414)
Palliative care management initiated at the time of the inclusion	216 (31.4%)
Median delay between initial diagnosis and palliative care initiation (months)	20.7 (0‐374)
Reasons for palliative care request	
Symptoms	161 (74.5%)
Psychological support	87 (40.3%)
Orientation of the patient	77 (35.6%)
Support for the patient's family	40 (18.5%)
Healthcare team support	24 (11.1%)
Terminal accompaniment	18 (8.3%)

17 (2.5%) patients have a caregiver and one dependent person at home.

a Tumor localizations accounting for at least 10% of the patients. ECOG‐PS: Eastern Cooperative Oncology Group Performance Status.

**Figure 2:**
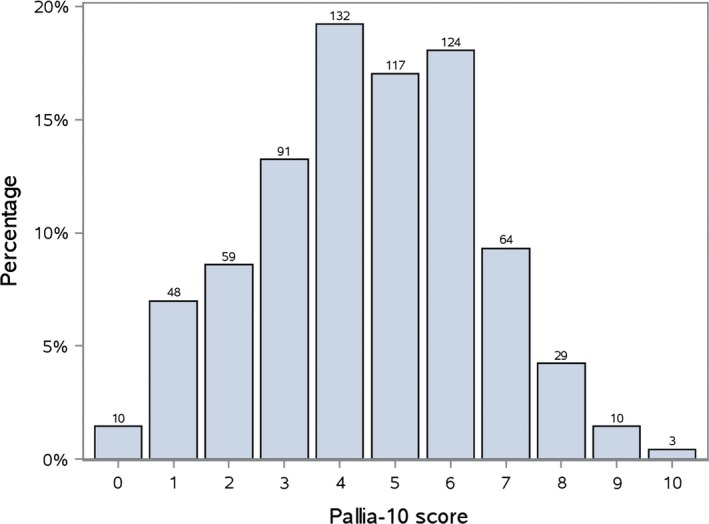
Distribution of PALLIA‐10 scores in the palliative population (n = 687). To note, ten palliative patients had a PALLIA‐10 scored 0

The PALLIA‐10 score distribution is presented in Figure 2. The prevalence of palliative patients with a PALLIA‐10 score greater than 3 in the population of patients hospitalized in a French cancer center is 479 (69.7%, 95%CI 66.1%‐73.1%), and the prevalence of palliative patients with a PALLIA‐10 score greater than 5 is 230 (33.5%, 95%CI 30.0%‐37.1%).

### 3.1 PC management

**Figure 3:**
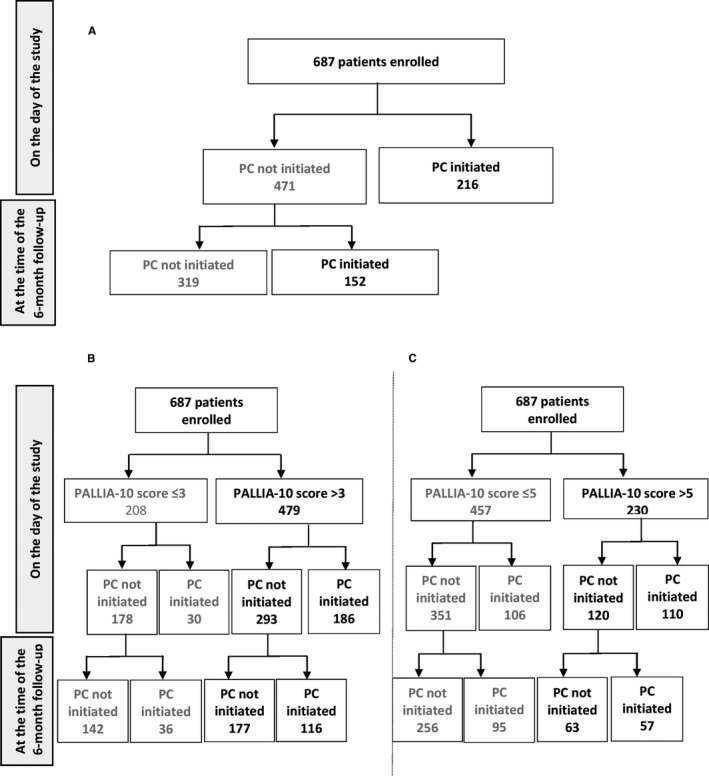
Distribution of the palliative care intervention in the palliative patient population at the time of the study and 6 months after the study (A), and distribution of the palliative patient population according to PALLIA 10 score ((B) score >3 and (C) score >5) and palliative care intervention at the time of the study and 6 months after the study

At the time of this study, 216 (31.4%) patients with a median PALLIA‐10 score of 6 (0‐9) already received PC. The PALLIA‐10 score was greater than 3 for 186 patients, ie 38.8% of the patients with a PALLIA‐10 score >3, and the PALLIA‐10 score was even greater than 5 for 110 patients, ie 47.8% of the patients with a PALLIA‐10 score >5. The 471 (69%) patients who were not receiving palliative care at the date of the study, had a median PALLIA‐10 score of 4 (0‐10). The PALLIA‐10 score was >3 for 293 patients, ie 61.2% of the patients with PALLIA‐10 score >3, and the PALLIA‐10 score was >5 for 120 patients ie 52.2% of the patients with PALLIA‐10 score >5 (Figure 3).

The initiation of PC started within the 6 months following the date of the study for 152 (22.1%) patients, who had a median PALLIA‐10 score of 4.5 (0‐10) at the time of the study, including 116 patients with a PALLIA‐10 score >3 (24.2% of the patients with a PALLIA‐10 score >3), and 57 with a PALLIA‐10 score >5 (24.8% of patients with a PALLIA‐10 score >5).

We explored factors potentially correlating with PC referrals, and results from the logistic regression analysis firstly lead to an adjusted odd ratio (OR) of 2.6 (CI95% 1.65‐4.11) for a PALLIA‐10 score >3 attesting that patients are significantly more often referred to a PC team when their PALLIA‐10 score was >3. Further exploration lead to an adjusted OR of 1.9 (1.17‐ 3.16) and 3.59 (2.18‐5.91) in the subpopulation of patients with PALLIA‐10 scored from 4 to 5, or scored at >5, respectively. The PALLIA‐10 score correlated with PC team intervention even after adjusting on number of metastatic sites, the convergence of opinions between the oncologist, the healthcare team and the PC team and reasons of hospitalization (Table 2).

**Table 2:** Predictive factors for palliative care team intervention. Final multivariate model of predictive factors of palliative care management

Variables		OR	95%CI	*P*‐value
PALLIA 10 Score	[0‐3] (Réf.)	1.00		<0.0001
	[3‐5]	1.924	[1.169‐3.165]	
	[5‐+]	3.595	[2.185‐5.914]	
Number of metastatic sites	No metastatic site (Réf.)	1.00		0.0294
	One metastatic site	0.426	[0.214‐0.846]	
	At least two metastatic sites	0.663	[0.356‐1.235]	
Opinion convergence (oncologist/health team/Palliative care team)	At least one disagree (Réf.)	1.00		0.0002
	All agree	3.942	[1.939‐8.017]	
Reasons of hospitalisation	Treatment (Réf.)	1.00		<0.0001
	Complications	2.731	[1.562‐4.777]	
	Symptoms	3.132	[1.949‐5.033]	

Predefined potential predictive factors were familial status, age at inclusion, reason for hospitalization, type of tumor, the opinion convergence between oncologist, healthcare team and palliative care team, Karnofsky score, number of metastatic sites, PALLIA 10 score (0‐3, 4‐5, 5‐+), and biological parameters (hemoglobin, lymphocytes, LDH, albumin, and CRP). Variables significant at a 20% level with less than 10% missing values in univariate analysis were used in a backward selection procedure to keep factors significant at a 5% level in the final predictive multivariate model.

The ROC analysis was performed and the PALLIA‐10 score defined as cut‐off point for optimal sensitivity and specificity was equal to 5 (Supplementary materials p4).

### 3.2 Overall survival (OS)

**Figure 4:**
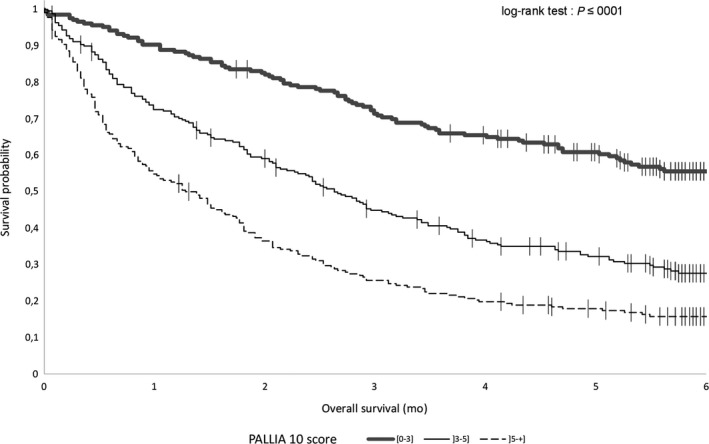
Overall survival. Kaplan‐Meier survival curves for patients with PALLIA‐10 score ≤3, 3 <PALLIA‐10 score ≤5, and PALLIA‐10 score >5 in the 687 palliative patients

The median OS for palliative patients was 2.73 months (95%CI, 2.43‐3.06). We explored the median OS in subpopulation of patients according to their PALLIA‐10 score. The median OS was not reached in the palliative patients with a PALLIA‐10 score ≤3. The median OS was 2.6 (95%CI, 2.1‐3.2) months in patients with a PALLIA‐10 score between 4 and 5, and 1.3 (95%CI, 0.95‐1.7) months in patients with a PALLIA‐10 score greater than 5 respectively (Figure 4).

The PALLIA‐10 score appeared as a reliable prognostic factor even after adjustment for Karnofsky index, reasons of hospitalization, type of tumor, the convergence of opinions between the oncologist, the healthcare team and the PC team, number of metastatic sites, and lymphocytes levels. PALLIA‐10 score between 4 and 5: HR = 1.58 (CI95% 1.20‐2.08), PALLIA‐10 score greater than 5: HR = 2.18 (95%CI 1.63‐2.92) (Table 3). Considering the PALLIA‐10 score as a quantitative variable, we noticed a significant gradient (HR = 1.18, 95%CI 1.11‐1.24) even after adjustment for the beforehand mentioned variables (Supplementary materials, p5‐6).

**Table 3:** Prognostic factors for OS. Final model of the multivariate Cox analysis of predefined potential prognostic factors (age at inclusion, reason for hospitalization, type of tumors, palliative care management at the time of inclusion, the convergence of opinions between the oncologist, the healthcare team and the palliative care team, Karnofsky score, number of metastatic sites, range of PALLIA 10 score (0‐3, 4‐5, and 5‐+), and biological parameters (hemoglobin, lymphocytes, LDH, albumin, and CRP) on OS

Variables		Hazard Ratio	95%CI	*P*‐value
PALLIA 10 Score	[0‐3] (Réf.)			<0.0001
	]3‐5]	1.582	[1.203‐2.082]	
	]5‐+]	2.181	[1.628‐2.923]	
Karnofsky Score	>50 (Réf.)			<0.0001
	≤50	2.084	[1.667‐2.606]	
Reasons of hospitalisation	Treatment (Réf.)			0.0002
	Complications	1.091	[0.806‐1.478]	
	Symptoms	1.597	[1.248‐2.044]	
Type of tumour	Breast (Réf.)			<0.0001
	Head and neck	1.747	[1.140‐2.678]	
	Bone and soft tissue	1.016	[0.641‐1.609]	
	Lung and pleural	1.939	[1.403‐2.682]	
	Digestive tract	1.662	[1.212‐2.279]	
	Gynecologic	0.894	[0.610‐1.310]	
	Urologic	1.100	[0.765‐1.582]	
	Others	0.908	[0.585‐1.410]	
Opinion convergence (oncologist/health team/Palliative care team	At least one disagree (Réf.)			0.0019
	All agree	1.672	[1.209‐2.312]	
Number of metastatic sites	No metastatic site (Réf.)			0.0003
	One metastatic site	0.802	[0.535‐1.200]	
	At least two metastatic sites	1.311	[0.886‐1.940]	
Lymphocytes	>0.7 G/l (Réf.)			0.0047
	≤0.7 G/l	1.337	[1.093‐1.636]	

Factors selected for the multivariate analysis (ie variables significant at a 20% level with less than 10% missing values in univariate analyses were included in a backward selection procedure to keep factors significant at a 5% level in the final Cox prognostic multivariate model).

## 4 DISCUSSION

To the best of our knowledge, PREPA‐10 is the first prospective multicentric study exploring the use of PALLIA‐10, a multidimensional questionnaire dedicated to help referring inpatients to palliative care. We prospectively used the PALLIA‐10 questionnaire and determined its subsequent scoring in a large series of hospitalized patients. Our findings support the use of the PALLIA‐10 questionnaire, as an ease‐of‐use and helpful instrument that should be further used to identify cancer inpatients the most in need of palliative care in daily practice in comprehensive cancer centers. The scoring system was rapid and compatible to standard hospital procedures, and seems to have been highly convenient to relay patients’ information during shifts in health care teams. The scores were promptly determined for all but one patient. The broad participation of 18 of the 20 French cancer centers provides a substantial strength that helps to ensure the generalizability of these results. The use of the questionnaire was welcomed in almost all the cancer centers, and its implementation was successfully carried out in all investigational sites by any caregiver of the health teams.

The absence of consensus on the definition of the palliative setting (from early to terminal status of an incurable disease) increased the complexity of implementing recommendations for palliative care management. The palliative status, based on the single opinion of the medical oncologist, usually led to inaccurate and over‐optimistic predictions of survival.^33^ Prognostic factors may help to inform decision making.^18^, ^23^, ^24^, ^25^, ^26^ In addition, the use of the surprise question is currently debatable. The *NCCN Clinical Practice Guidelines in Oncology for Palliative Care* had been published, but the referral criteria from the two‐step screening were too sensitive for use as an automatic trigger; a one‐step questionnaire has been proposed to identify unmet palliative care needs.^30^, ^31^, ^32^ The PALLIA‐10 questionnaire was recommended in 2010 in standard practice by the SFAP in France, however supportive data proving the relevance of the PALLIA‐10 questionnaire were lacking to convince the palliative teams to use this innovative tool notwithstanding the recognized need for a decision‐making instrument to initiate PC, and the PALLIA‐10 questionnaire is not routinely used so far.^34^ However, this easy‐to‐use PALLIA‐10 form and its related score provide the advantage to address different complementary aspects in a context in which self‐assessment is tricky. Through an innovative multidimentional approach and an adapted semiology, the PALLIA‐10 questionnaire help to determine whether a dedicated palliative care intervention is timely required, decision which is reinforced by the hetero‐evaluation driven by health care referents other than medical oncologists themselves.

The major asset of the PALLIA‐10 questionnaire was to address key issues not only including clinical and biological criteria, but also psycho‐sociocultural and ethic aspects. This global approach while partly subjective, is coherent with palliative care aims and philosophy, and our study shows that it is also statistically robust. We strongly support the use of such a score, easily achieved through the use of a single, simple to implement, multidimensional questionnaire. We did not intend to set up a psychometric validation of this questionnaire; some of its criteria are currently explored through various questionnaires, and despite further validation of its content as a whole is needed, the PALLIA‐10 score appeared as a predictive factor to refer patients to PC team intervention. The PALLIA‐10 criteria still warrant an in‐depth qualitative analysis to determine the impact of each criteria and especially determine through multidimentional aspects of the questionnaire, the added value of the innovative psychosocial criteria.

The present results highlight that the predefined threshold score >3 and >5 were consistent. However, a score greater than 3 as currently recommended would refer too many patients according to the current capacity of PC teams in the specific context of comprehensive cancer centers, and this series, strengthened by a ROC analysis, shows that a score greater than 5 would be more appropriate. We reported that patients with a high PALLIA‐10 score are more often supported by PC management even after adjusting for (1) metastatic status, (2) convergence of opinions between the oncologist, the healthcare team, and the PC team, and (3) the reasons of hospitalization. In addition, the PALLIA‐10 score appeared as a reliable prognostic factor for death at 6 months, independent from the variation of other severity criteria such as Karnofsky index, reasons of hospitalization, type of tumor, number of metastatic sites, the convergence of opinions between the oncologist, the healthcare team and the PC team, and lymphocyte count. Interestingly, we noted an increased relevance of the PALLIA‐10 score when considered as a quantitative variable, leading to a significant gradient (adjusted HR = 1.18, 95%CI 1.11‐1.24) associating one unit increase in the score with a reduced overall survival.

In our series, we identified 82% of inpatients in palliative setting that all should be followed‐up by a PC team. In the global population, 216 (31%) patients were already followed‐up by a dedicated PC team at the time of this survey with a median PALLIA‐10 score reached 6 (0‐9). The PALLIA‐10 score was greater than 3 for 479 (70%) patients for whom referring to PCs should be primarily performed. However, only 186/479 received palliative care. Even if we recognize and agree with this need of PC management, the current size of the PC teams are unfortunately not yet adapted to support as many patients, and no major development can be foreseen in the coming years. The reluctance to admit advanced stage or progression of the disease results in a very late referral to the PC teams.^35^, ^36^ Many barriers to referral to PC still exist in comprehensive centers in France and implementation of the PALLIA‐10 questionnaire could help lead changing this. In parallel, it is critical to reinforce the training in PC of medical teams (oncologists and nurses) to complement the interventions of specialized PC team.

Our study has several limitations. Firstly, this series focusing on cancer patients from comprehensive cancer centers does not allow general applicability. In addition, the study focused on adult inpatients, and surgery patients were excluded. Indeed, while we acknowledge that some of them can benefit from early palliative care, they are probably few and we decided to focus on medical oncology patients and their caregivers who are currently our main target. Furthermore, it should be underlined that the role of PC must not be exclusively restricted anymore to late‐stage interventions or hospitalized patients.^6^ PC teams should simultaneously not neglect their valuable role in outpatient hospitalization and improve quality of life of cancer patients through early PC intervention.^37^, ^38^

Therefore, the PALLIA‐10 threshold value >5 already observed in more than one‐third of palliative patients appeared more consistent and more realistic in the specific context of the current abilities of PC teams in French comprehensive cancer center. Further randomized studies deserve to evaluate the impact of PALLIA‐10 score implementation on patients referred to palliative teams, to explore quality of life, and to appraise in parallel the relief of suffering of the caregivers by limiting the occurrence of unbalanced events.

The PALLIA‐10 score with medical and psychosocial criteria is an interesting decision assistance‐tool for PC management initiation and a prognostic indicator for OS. However, the threshold at 3 appeared too sensitive for patients hospitalized in comprehensive centers, and the current resources allocated to palliative teams. A threshold at 5 should allow the identification of patients to absolutely refer to palliative teams. Earlier stage intervention should in parallel be reinforced by expanding intervention in/through consultation and outpatient hospitalization. Qualitative aspects and evaluation of the impact of constitutive items of the questionnaire warrant to be explored with special expectation on innovative criteria impacts such as psycho‐sociological issues for a better use of this palliative care needs assessing scale.

## CONFLICT OF INTEREST

The authors declared no potential conflict of interest with respect to the research, authorship and/or publication of this article.

## AUTHOR CONTRIBUTIONS

Yann MOLIN: Conceptualization, Funding acquisition, Investigation, Methodology, Writing original draft, and review; Caroline GALLAY: Investigation, Writing original draft, review; Julien GAUTIER: Conceptualization Data curation Funding acquisition Methodology Project administration resources, software, supervision, Writing ‐ original draft, and writing ‐ original draft, review; Audrey LARDY‐CLEAUD: Formal analysis, writing ‐ original draft, review; Romaine MAYET: Data curation, project administration, resources, software, supervision, writing ‐ original draft, review; Marie‐Christine GRACH: Investigation, Writing original draft, review; Gérard GUESDON: Investigation, Writing original draft, review; Géraldine CAPODANO: Investigation, Writing original draft, review; Olivier DUBROEUCQ: Investigation, Writing original draft, review; Carole BOULEUC: Investigation, Writing original draft, review; Nathalie BREMAUD: Investigation, Writing original draft, review; Anne FOGLIARINI: Investigation, Writing original draft, review; Aline HENRY: Investigation, Writing original draft, review; Nathalie CAUNES‐HILARY: Investigation, Writing original draft, review; Stéphanie VILLET: Investigation, Writing original draft, review; Christine VILLATTE Investigation, Writing original draft, review; Véronique FRASIE: Investigation, Writing original draft, review; Valérie TRIOLAIRE Investigation, Writing original draft, review; Véronique BARBAROT: Investigation, Writing original draft, review; Jean‐Marie COMMER: Investigation, Writing original draft, review; Agnès HUTIN: Investigation, Writing original draft, review; Gisèle CHVETZOFF: Conceptualization, Funding acquisition, Investigation, Methodology, supervision Writing original draft, review and editing.

All authors reviewed, discussed, and agreed to their individual contributions prior to submission.
