# Supplementary material for: PALLIA‐10, a screening tool to identify patients needing palliative care referral in comprehensive cancer centers: A prospective multicentric study (PREPA‐10)
Source: Cancer Med. 2019 May 4;8(6):2950–61. doi: 10.1002/cam4.2118 (PMC6558580; doi:10.1002/cam4.2118)
Supplement: Supplementary file 1 [file CAM4-8-2950-s001.pdf]

Appendices/Supplemental Material

Content page: Supplementary Tables.

Supplementary S1. The PALLIA 10 questionnaire. A. Translated version of the PALLIA-10 questionnaire, B. Original version of the PALLIA-10 questionnaire …………………..………................. p1-2

Supplementary S2. Recruiting sites, investigators, and numbers of inclusions at each recruiting sites………………………………………………………………………………………… p3

Supplementary S3. Receiver operator characteristic (ROC) analysis of the PALLIA-10 scores. The Youden index J was calculated to determinate the cut-off point for optimal sensitivity and specificity….. p4

Supplementary S4. Overall survival. Kaplan meier survival curves for patients according to their by PALLIA-10 score ..……..………………………………… …………...………………...…… p5

Supplementary S5. Final model of the multivariate Cox analysis………….........……………………….. p6

Supplementary S1. **The PALLIA 10 questionnaire.** A. Translated version of the PALLIA-10 questionnaire, B. Original version of the PALLIA-10 questionnaire http://www.sfap.org/rubrique/pallia-10, p2)

A.

When should a palliative care team be requested? 10 questions survey- a decision assistance-tool.

Access to palliative care:

- A right for patients
- A professional obligation for health care teams

	Questions	Additional comments	Yes/No
1	The patient is currently suffering from an incurable disease at the present state of knowledge	A positive response is a necessary condition to start using the PALLIA 10 questionnaire and to answer the following questions	
2	There are negative prognostic factors	Factors that have been validated in oncology: hypoalbuminemia, inflammatory syndrome, lymphopenia, Performance Status >3 or corresponding Karnofsky index	
3	The disease is a rapidly progressive disease		
4	Patients or his/her caregiver who are seeking palliative care and support	French law n°99-477 dated June 9th 1999 seeks to guarantee individuals their rights to access palliative care	
5	Persistent and unrelieved symptoms that remain despite the use of first-intent treatment	Spontaneous or care-induced pain: dyspnoea, vomiting, occlusive syndrome, confusion, agitation etc…	
6	You note psychological frailty factors in the patient and/or his/her neighbourhood	Sadness, anxiety, inward-looking attitudes, aggressiveness, behavioural troubles, communication troubles, family conflicts, pre-existing psychopathology in the patient and his/her neighbourhood	
7	You note psychological frailty factors in the patient and/or his/her caregiver	Isolation, precarious situation, physical dependence, impact of health care, financial difficulty, presence of a dependant person in neighbourhood, young children	
8	The patient or his/her neighbourhood have difficulties to assimilate information on the disease and/or its prognosis	Facing anxiety induced by disease progression, the patients and/or his/her caregiver setting up psychological defense mechanisms making the communication difficult and the initiation of palliative care more complicated	
9	You observe questions and/or divergences within the health-care team regarding the consistency of the care plan	These questions may concern:<br>• Anticipated prescriptions<br>• Indication: hydration, feeding, antibiotherapy, catheter insertion, transfusion, surveillance (glycemia, monitoring…)<br>• Indication and initiation of sedation<br>• Service at the point of care<br>• Critical care status	
10	You are wondering about the appropriate approach regarding:<br>• Refusal of treatment<br>• Limitation or discontinuation of treatment<br>• Request for euthanasia<br>• Conflicts of values	The Leonetti's French law relative to patients' right and the end of life setting out issues relative to the refusal of treatment and modalities of decisions for treatment discontinuation and limitation in patients who are able or not to express their willingness.	

Please answer each question. "Yes" = 1 point

In the event of three or more positive responses, access to a specialized palliative care team should be considered.

B.

	Questions	Complément	Oui/Non
1	Le patient est atteint d'une maladie qui ne guérira pas, en l'état actuel des connaissances	Une réponse positive à cette question est une condition nécessaire pour utiliser Pallia 10 et passer aux questions suivantes	
2	Il existe des facteurs pronostiques péjoratifs	Validés en oncologie : hypo albuminémie, syndrome inflammatoire, lymphopénie, Performans Status >3 ou Index de karnofsky	
3	La maladie est rapidement évolutive		
4	Le patient ou son entourage sont demandeurs d'une prise en charge palliative et d'un accompagnement	Loi n° 99-477 du 9 juin 1999 visant à garantir le droit d'accès aux soins palliatifs	
5	Il persiste des symptômes non soulagés malgré la mise en place des traitements de première intention	Douleur spontanée ou provoquée lors des soins, dyspnée, vomissements, syndrome occlusif, confusion, agitation …	
6	Vous identifiez des facteurs de vulnérabilité d'ordre psychique pour le patient et/ou son entourage	Tristesse, angoisse, repli, agressivité ou troubles du comportement, troubles de la communication, conflits familiaux, psycho- pathologie préexistante chez le patient et son entourage	
7	Vous identifiez des facteurs de vulnérabilité d'ordre social chez le patient et/ou l'entourage	Isolement, précarité, dépendance physique, charge en soins, difficultés financières, existence dans l'entourage d'une personne dépendante, enfants en bas âge	
8	Le patient ou l'entourage ont des difficultés d'intégration de l'information sur la maladie et/ou sur le pronostic	Face à l'angoisse générée par la maladie qui s'aggrave, les patients, l'entourage peuvent mettre en place des mécanismes de défense psychologique qui rendent la communication difficile et compliquent la mise en place d'un projet de soin de type palliatif	
9	Vous constatez des questionnements et/ou des divergences au sein de l'équipe concernant la cohérence du projet de soin	Ces questionnements peuvent concerner :<br>• prescriptions anticipées<br>• indication : hydratation, alimentation, antibiothérapie, pose de sonde, transfusion, surveillance du patient (HGT, monitoring …)<br>• indication et mise en place d'une sédation<br>• lieu de prise en charge le plus adapté<br>• statut réanimatoire	
10	Vous vous posez des questions sur l'attitude adaptée concernant par exemple :<br>• un refus de traitement<br>• une limitation ou un arrêt de traitement<br>• une demande d'euthanasie<br>• la présence d'un conflit de valeurs	La loi Léonetti relative au droit des malades et à la fin de vie traite des questions de refus de traitement et des modalités de prise de décisions d'arrêt et de limitation de traitement autant chez les patients compétents que chez les patients en situation de ne pouvoir exprimer leur volonté	

Supplementary S2. **Recruiting sites, investigators, and numbers of inclusions at each recruiting sites.**

Name of the principal investigator* and recruiting co-investigators	Investigation site	Number of patients / investigation site
CHVETZOFF Gisèle,* MOLIN Yann	Centre Léon Bérard	100
GALLAY Caroline	Institut Régional du Cancer de Montpellier	73
GRACH Marie-Christine	Centre François Baclesse	67
GUESDON Gérard	Institut Bergonié	64
CAPODANO Géraldine	Institut Paoli Calmettes	60
DUBROEUCQ Olivier	Institut Jean Godinot	53
BOULEUC Carole	Institut Curie – Paris	50
BREMAUD Nathalie	Centre Georges-François Leclerc	49
FOGLIARINI Anne	Centre Antoine Lacassagne	44
HENRY Aline	Institut de Cancérologie de Lorraine	42
CAUNES-HILARY Nathalie	IUC Toulouse Oncopole	40
VILLET Stéphanie	Centre Oscar Lambret	40
VILLATTE Christine	Centre Jean Perrin	37
FRASIE Véronique	Centre Paul Strauss	34
TRIOLAIRE Valérie	Institut Curie - Saint Cloud	30
BARBAROT Véronique	ICL Nantes	25
COMMER Jean-Marie	ICL Angers	18
HUTIN Agnès	Centre Eugène Marquis	14

Supplementary S3. Receiver operator characteristic (ROC) analysis of the PALLIA-10 scores. The Youden index J was calculated to determinate the cut-off point for optimal sensitivity and specificity.

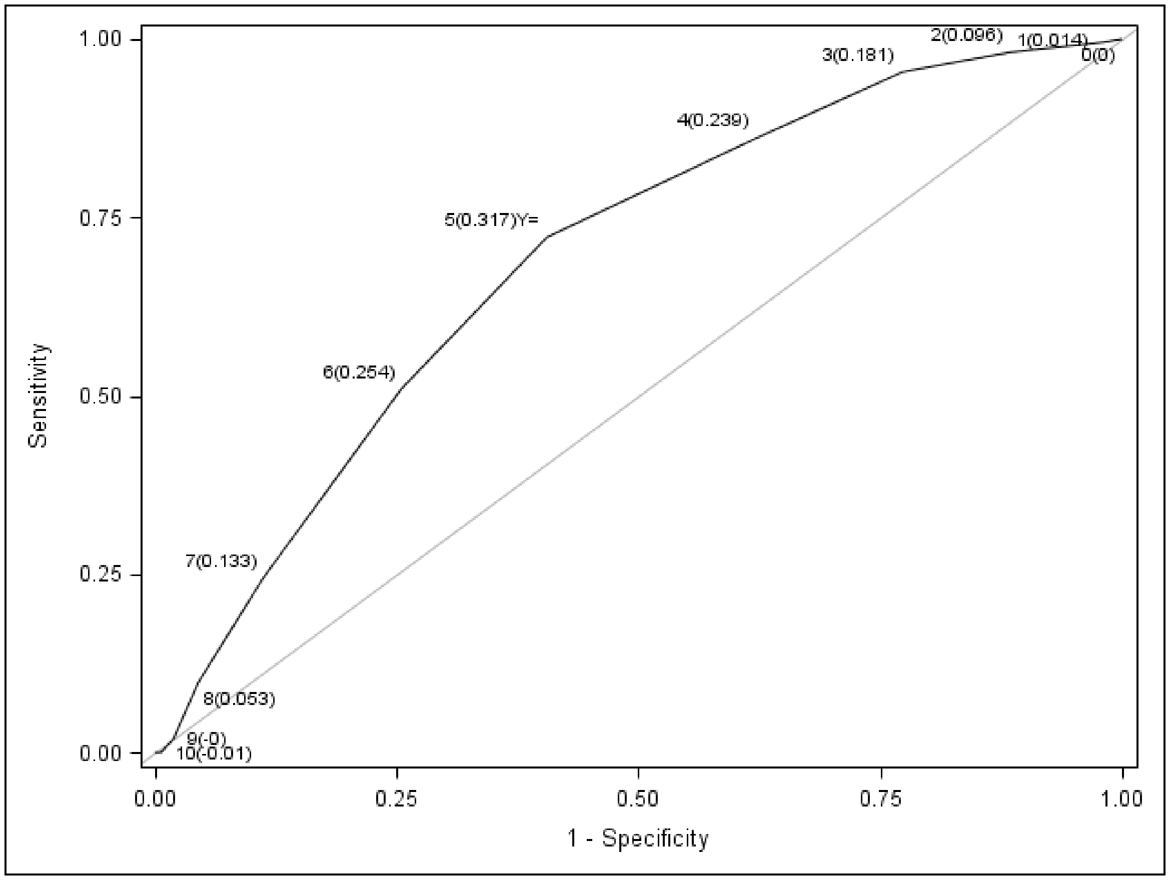

Supplementary S4, **Figure S**. **Overall survival.** Kaplan meier survival curves for patients according to their PALLIA-10 score.

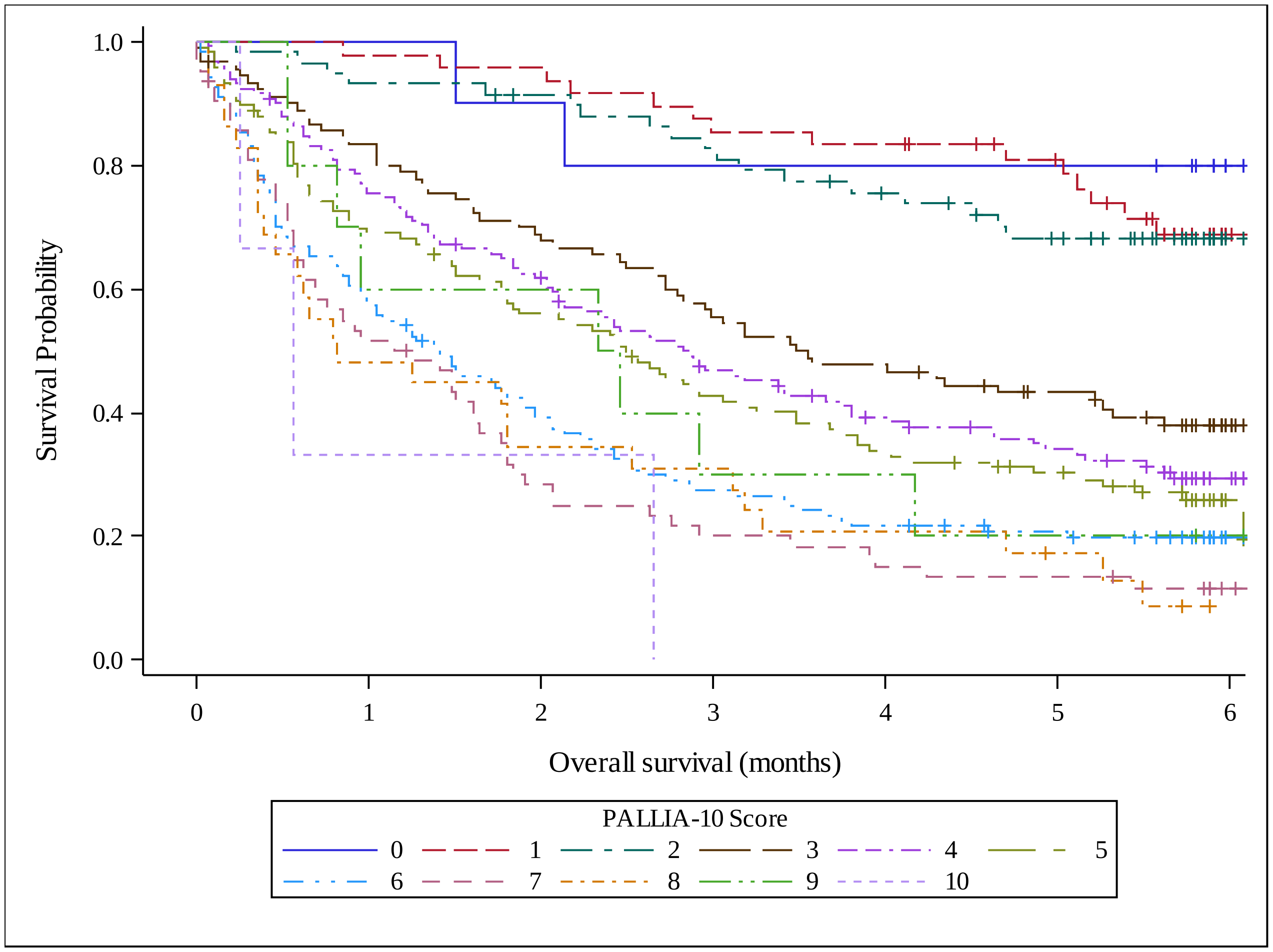

Supplementary S5. Final model of the multivariate Cox analysis (PALLIA-10 score as quantitative variable).

Variables		Hazard Ratio	[CI 95%]	P-value
PALLIA-10 score	Quantitative value	1.175	[1.110-1.244]	<.0001
Karnofsky score	>50 (Réf.)			<.0001
	≤50	1.995	[1.589-2.504]	
Reasons of hospitalization	Treatment (Réf.)			<.0001
	Complications	1.092	[0.807-1.479]	
	Symptoms	1.622	[1.269-2.074]	
Tumor localization	Breast (Réf.)			<.0001
	Head and neck	1.731	[1.132-2.647]	
	Bone and soft tissue	0.995	[0.629-1.574]	
	Lung and pleural tumors	1.991	[1.442-2.748]	
	Digestive system	1.719	[1.251-2.362]	
	Gynecological	0.837	[0.571-1.227]	
	Urological	1.110	[0.771-1.596]	
	Others	0.926	[0.597-1.438]	
Number of metastases	No metastatic site (Réf.)			0.0004
	One metastatic site	0.762	[0.510-1.140]	
	At least two metastatic sites	1.230	[0.831-1.820]	
Lymphocyte rate	>0.7 G/l (Réf.)			
	≤ 0.7 G/l	1.350	[1.103-1.652]	0.0036
