# Supplementary figures and images for: PALLIA‐10, a screening tool to identify patients needing palliative care referral in comprehensive cancer centers: A prospective multicentric study (PREPA‐10)
Source: Cancer Med. 2019 May 4;8(6):2950–61. doi: 10.1002/cam4.2118 (PMC6558580; doi:10.1002/cam4.2118)

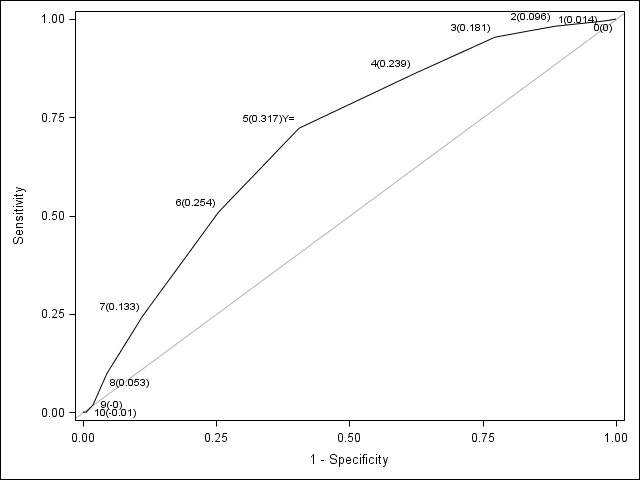

Supplement: Supplementary file 2 [file CAM4-8-2950-s002.tiff]

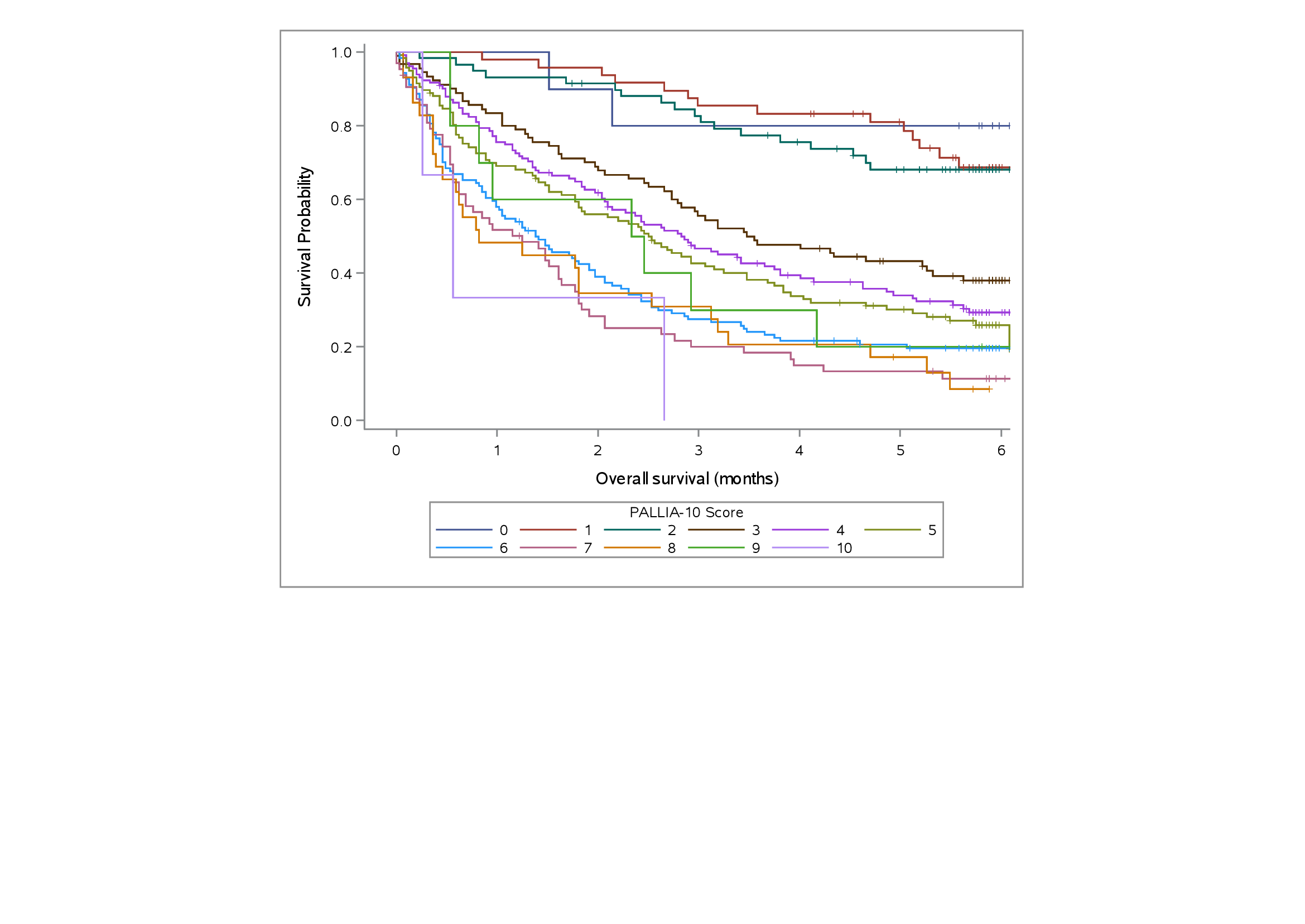

Supplement: Supplementary file 3 [file CAM4-8-2950-s003.tiff]
